# The treatment of tuberculosis in the upper thoracic spine using the small incision technique through the third rib

**DOI:** 10.3389/fsurg.2023.1236611

**Published:** 2023-09-05

**Authors:** Jibin Ma, Zepei Zhang, Jie Lan, Jiwei Tian, Fulin Chen, Jun Miao

**Affiliations:** ^1^ Department of Spine Surgery, Tianjin Hospital, Tianjin University, Tianjin, China; ^2^ Department of Orthopedics, The Second People’s Hospital of Changzhi, Changzhi, China; ^3^ Department of Orthopedics, Chu Hisen-I Memorial Hospital of Tian jin Medical University, Tianjin, China

**Keywords:** tuberculosis, spine, upper thoracic spine, spinal fusion, minimally invasive surgery

## Abstract

**Background:**

The complex anatomical structure of the upper thoracic spine makes it challenging to achieve surgical exposure, resulting in significant surgical risks and difficulties. Posterior surgery alone fails to adequately address and reconstruct upper thoracic lesions due to limited exposure. While the anterior approach offers advantages in fully exposing the anterior thoracic lesions, the surgical procedure itself is highly intricate. Although there exist various anterior approaches for the upper thoracic spine, the incidence of upper thoracic spine lesions is relatively low. Consequently, there are limited reports on the treatment and reconstruction of upper thoracic spine lesions using the third rib small incision approach in the context of upper thoracic tuberculosis.

**Methods:**

We collected data from four patients with upper thoracic tuberculosis who were admitted to our department between July 2017 and November 2022. The treatment for upper thoracic tuberculosis involved utilizing the third rib small incision approach, which included two cases of thoracic 3–4 vertebral tuberculosis, one case of thoracic 4 vertebral tuberculosis, and one case of thoracic 5 vertebral tuberculosis. Among the patients, three were positioned in the left lateral position, while one was positioned in the right lateral position. Prior to admission, all four patients received a two-week course of oral medication, consisting of isoniazid, rifampicin, pyrazinamide, and ethambutol. After the surgical procedure, they continued receiving anti-tuberculosis treatment for a duration of 12 months.

**Results:**

The average duration of the surgical procedure was 150 min, with an average blood loss of 500 ml. One patient exhibited symptoms of brachial plexus injury, which gradually improved after careful observation. All patients experienced primary wound healing, and no complications such as pulmonary infection, respiratory failure, or other adverse events were observed. Additionally, one patient showed elevated transaminase levels, leading to a modification in the anti-tuberculosis drug regimen from quadruple therapy to triple therapy.

**Conclusion:**

The treatment of upper thoracic tuberculosis through the third rib small incision technique is a very good surgical approach, which has the advantages of safety and effectiveness.

## Introduction

Tuberculosis remains a persistent public health concern, warranting continuous attention. The transmission of tuberculosis occurs primarily through the respiratory tract, as well as through the dissemination of mycobacteria via the blood or lymphatic system to other parts of the body. Among extrapulmonary tuberculosis cases, spinal tuberculosis is a commonly encountered form (1), with thoracic tuberculosis ranking second in terms of incidence, following lumbar tuberculosis, within the spectrum of spinal tuberculosis. As the drug resistance rate of Mycobacterium tuberculosis rises, the incidence of spinal tuberculosis is also increasing. Extrapulmonary tuberculosis can be managed through conservative or surgical approaches. Mild cases may be suitable for conservative treatment, whereas surgical intervention is recommended for patients with extensive abscesses and neurological symptoms. The progression of thoracic tuberculosis can lead to kyphosis and neurological damage in affected individuals, with severe cases carrying the risk of paraplegia.

According to the 2020 global tuberculosis report published by the World Health Organization (WHO) (2): China ranks third worldwide in terms of tuberculosis cases, accounting for 8.4% of total cases. India (26.0%) and Indonesia (8.5%) hold the first and second positions, respectively. Literature indicates that extrapulmonary tuberculosis accounts for 10%–15% of all tuberculosis cases, with skeletal system involvement, particularly in the spine, ranking second only to lymph nodes (3). In fact, spinal tuberculosis represents the most prevalent form of bone and joint tuberculosis, comprising approximately 50% of such cases (4).

The complex anatomical structure of the upper thoracic spine poses challenges in surgical exposure, with a high degree of risk and difficulty. Posterior surgery alone is insufficient to fully address and reconstruct upper thoracic lesions due to limited exposure. While anterior surgery offers certain advantages, it is accompanied by a highly intricate surgical approach. Limited research has been conducted on the utilization of anterior and lateral anterior approaches for the treatment and reconstruction of upper thoracic lesions. The occurrence of tuberculosis in the upper thoracic vertebra (T1-T4) is relatively rare, attributed to its unique anatomical location and rich blood supply. Consequently, there is limited literature available on this specific topic. In our study, we employed the third rib small incision approach to successfully treat upper thoracic tuberculosis, yielding satisfactory recent outcomes. In this paper, we will delve into the surgical approach and the selection of the surgical procedure.

### Case presentation

#### General information

From July 2017 to November 2022, the Tianjin Hospital of Tianjin University treated four patients with upper thoracic tuberculosis. Patients provide their informed consent in accordance with applicable regulations and seek approval from the Ethics Committee. Among them, there were three males and one female, with an average age of 41.5 years. The cases included two instances of thoracic 3–4 vertebral tuberculosis, one case of thoracic 4 vertebral tuberculosis, and one case of thoracic 5 vertebral tuberculosis. The time from the onset of symptoms to the diagnosis for all patients ranged from 1 to 3 months. The primary complaints were chest and back pain. Additionally, the patients experienced symptoms of tuberculosis poisoning, such as fatigue, night sweats, weight loss, and afternoon fever. Upon admission, examinations revealed mild kyphosis of the thoracic spine, mild tenderness of the spinous process of the thoracic spine, noticeable percussion pain, limited thoracic spine mobility, no radiating pain in both lower limbs, no evident abnormalities in the cervical and lumbar spine, normal sensation in both lower limbs, V-grade muscle strength in both lower limbs, negative results in the straight leg raising test and strengthening test of both lower limbs, negative bilateral abdominal wall reflex, knee tendon reflex, Achilles tendon reflex, negative bilateral Hoffmann sign, and negative Babinski sign. Laboratory examinations revealed mild anemia in one patient upon admission, with elevated erythrocyte sedimentation rate and C-reactive protein levels. Prior to the operation, all four patients were administered oral anti-tuberculosis drugs, including isoniazid, rifampicin, pyrazinamide, and ethambutol, for a period of two weeks.

#### Surgical indications

The abscess is situated at levels T1–4 and is confined to the anterior region. Moreover, the patient exhibits a generally favorable health condition, indicating their suitability and capacity to withstand the surgical procedure.

#### Surgical contraindications

The presence of the abscess in the lower thoracic vertebrae, combined with the patient’s compromised physical condition, renders them unsuitable for undergoing surgical intervention.

#### Surgical approach

**Figure 1:**
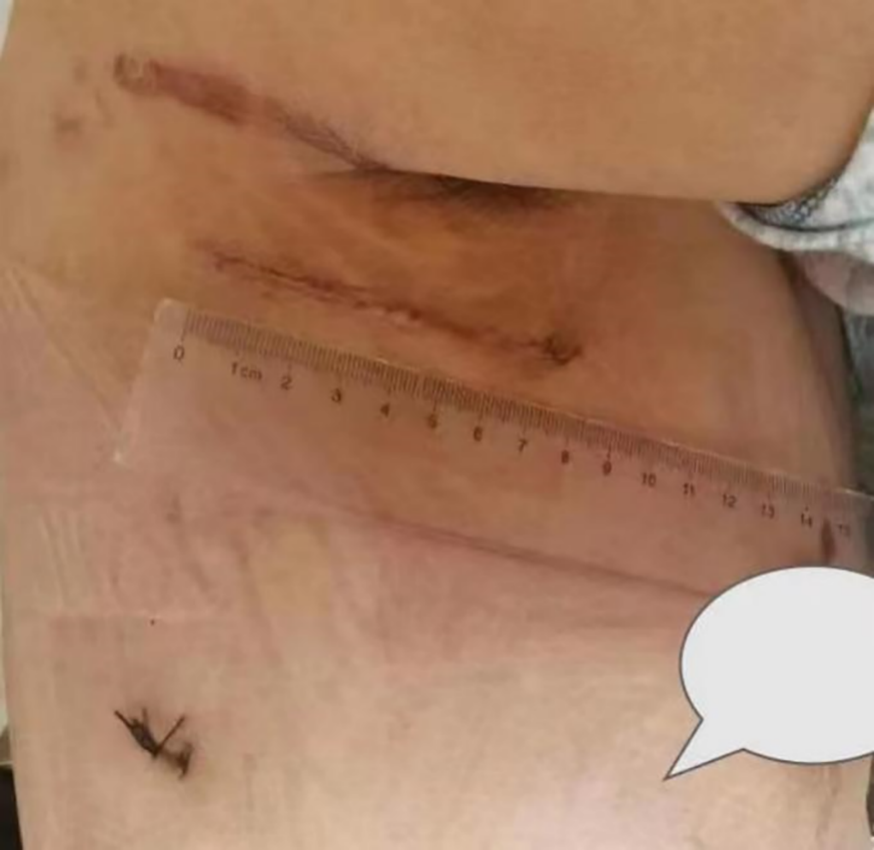
Photograph of the surgical incision appearance.

General anesthesia and double lumen intubation were administered for the procedure. The choice of surgical approach was determined based on the direction and location of the abscess. Three patients underwent the left approach, while one patient underwent the right approach. The patient was positioned laterally, with the upper limb abducted at 135 degrees and flexed at 45 degrees. To ensure stability, the patient's upper limb was fixed using an upper limb abduction bracket. On the outer side of the subaxillary thorax, a line approximately 8 cm–10 cm in length was marked along the surface of the third rib (Figure 1). The posterior end of the line extended to the outer edge of the scapula, while the anterior end reached the outer edge of the pectoralis major muscle.The surgical procedure involved a sequential dissection of the skin, subcutaneous tissue, and deep fascia. The posterior aspect of the incision extended to the anterior edge of the latissimus dorsi muscle, which may come into contact with the thoracodorsal artery located beneath it. If necessary, the artery could be ligated to provide better exposure of the third rib. By palpating the third rib, the intercostal muscle was incised along its course, allowing for the liberation and subsequent removal of the rib within the length of the incision. This exposed the parietal pleura, which was then incised to enter the pleural cavity and deflate the lung. A gentle traction with a chest opener facilitated the opening of the chest wall, revealing the heart and aortic arch in cases following the left approach. Careful manipulation of the aortic arch and heart allowed for their anterior displacement, exposing the left side of the vertebral body. The right approach, on the other hand, did not encounter obstruction from the aortic arch, and by cautiously pulling the heart forward, the right side of the vertebral body could be exposed. Intraoperative statistics indicated that the four patients in this group had exposure spanning from the thoracic 2nd to the 7th vertebrae. Longitudinal incisions were made on the parietal pleura adjacent to the vertebral body. The segmental vessels associated with the affected vertebral body, as well as those of the upper and lower vertebral bodies, were meticulously ligated and excised.

#### Surgery method

**Figure 2:**
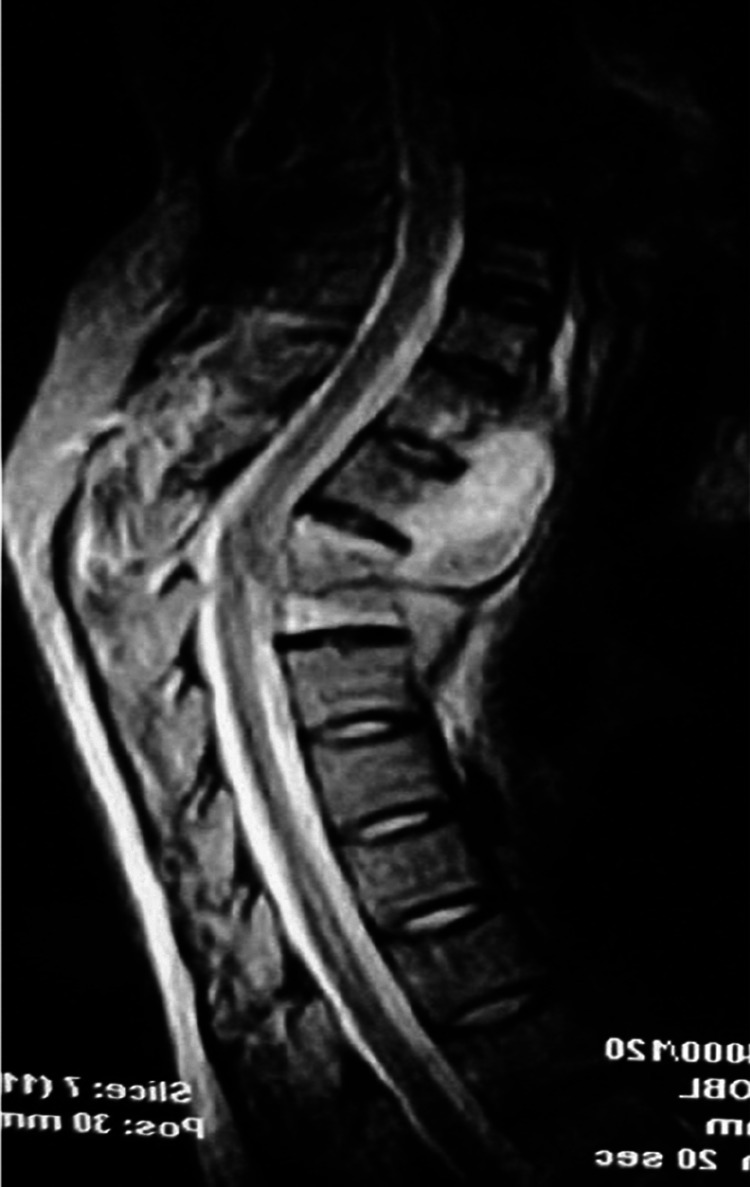
T3–4 vertebral tuberculosis.

**Figure 3:**
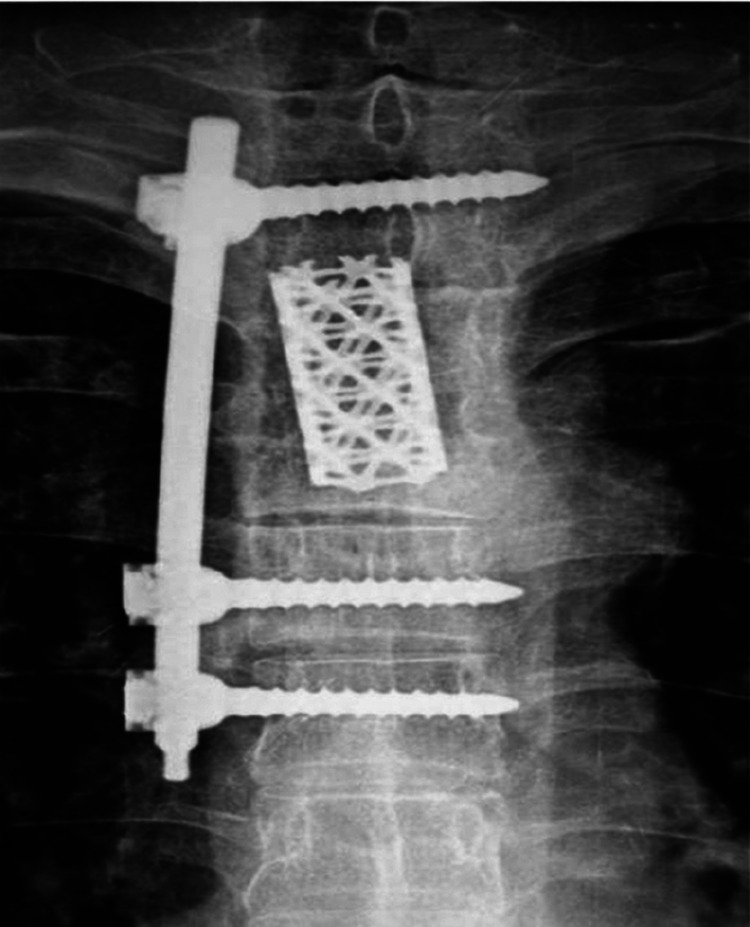
Postoperative pa x-ray.

**Figure 4:**
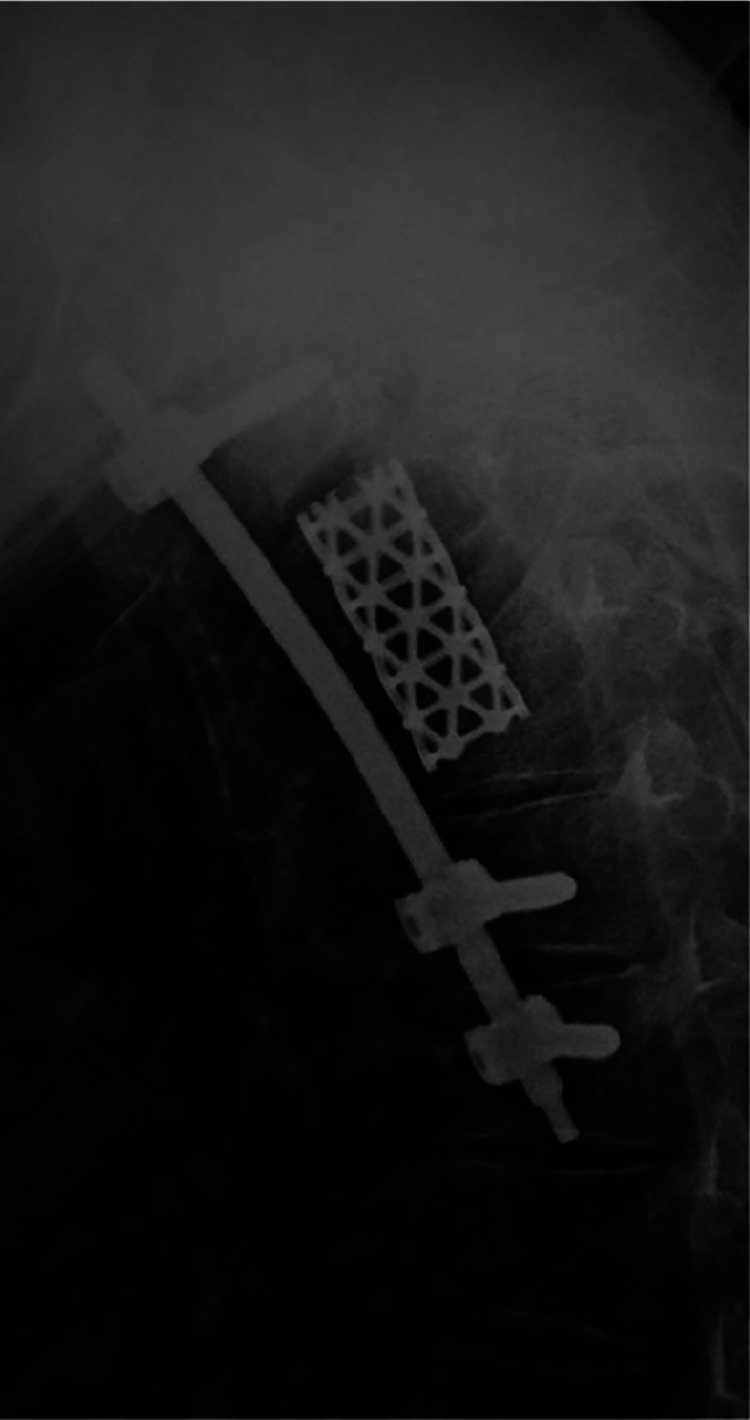
Postoperative lat x-ray.

In patients with thoracic 3–4 vertebral tuberculosis, the affected vertebral bodies (T3–T4) were surgically removed. Subsequently, a titanium mesh was used for bone grafting, and a nail rod system was fixed onto the adjacent vertebral bodies, namely thoracic 2, 5, and 6 (The DePuySynthes Expedium 5.5 pedicle screw system has a diameter of 5.0–6.0 mm and a depth of 35–40 mm, Figure 2–4). Similarly, in patients with tuberculosis affecting the thoracic 4 vertebral body, the affected vertebra was removed and replaced with a titanium mesh-supported bone graft. Additionally, a titanium plate was fixed onto the adjacent thoracic 3 and 5 vertebral bodies. In cases of tuberculosis involving the thoracic 5 vertebral body, the affected vertebra was excised and replaced with a titanium mesh-supported bone graft. A titanium plate was then fixed onto the neighboring thoracic 4 and 6 vertebral bodies (The Anterior Thoracic titanium Plate is the FXY-A Anterior Thoracic Plate made in China). The bone graft was obtained from the third rib, and a closed thoracic drainage tube was placed in the posterior axillary line between the 7th and 8th ribs. Following thorough rinsing of the chest cavity and inflation of the lungs, the chest was meticulously closed layer by layer.

#### Postoperative treatment

After the operation, the patient's head was elevated and the foot was positioned lower, which facilitated thoracic drainage. The drainage volume was less than 50 ml per 24 h. The patients received broad-spectrum antibiotics for 48 h, perioperative analgesia, and anti-tuberculosis treatment. The anti-tuberculosis regimen consisted of isoniazid, rifampicin, pyrazinamide, and ethambutol for 3 months, followed by oral isoniazid and rifampicin for 9 months, with a total treatment duration of 12 months. Standard preventive measures were implemented to reduce the risk of deep vein thrombosis after the operation. Following the removal of the drainage tube, a brace was worn for stabilization. Activities involving spinal torsion and heavy lifting were avoided for 4–6 weeks to promote successful bone graft fusion. x-ray examinations were conducted at 3 months, 6 months, and 1 year post-operation. Liver and kidney function were monitored monthly during the recovery period. One patient experienced elevated transaminase levels for 2 months, and after receiving liver protection treatment, the patient's regimen was adjusted to triple therapy with isoniazid, rifampicin, and ethambutol. Nutritional support was enhanced throughout the recovery process.

## Results

The average duration of the operation was 150 min, with an average blood loss of 500 ml. One patient who underwent surgery for thoracic 3–4 vertebral tuberculosis experienced symptoms of brachial plexus injury, likely resulting from the high incision position and compression of the brachial plexus during thoracotomy. However, these symptoms gradually improved and returned to normal within 2 weeks.

The postoperative follow-up period extended from 3 to 24 months, with an average duration of 18 months. Notably, callus growth was observed merely 2 months after the surgical intervention, and complete bone healing was successfully attained within 3–6 months post-surgery. Regular postoperative x-ray evaluations showed stable internal fixation without any signs of displacement in all patients. Furthermore, patients with upper thoracic tuberculosis experienced relief from postoperative chest and back pain.

## Discussion

The surgical approach for treating upper thoracic tuberculosis varies depending on the specific conditions of each patient and the chosen surgical methods. Common surgical approaches include the following: the first is anterior surgery: This approach involves accessing the thoracic region through the front of the chest, typically through the supraclavicular or substernal approach. It is suitable for patients with fewer pulmonary complications. The senond is posterior surgery: This approach involves accessing the spine through the back. Common posterior surgical techniques include unilateral posterior, bilateral posterior, and expanded posterior approaches. This approach is suitable for patients with spinal cord compression, chest wall tumors, disc herniation, and other specific circumstances. The third is comprehensive approach: Depending on the patient's specific conditions and surgical requirements, a combination of multiple surgical approaches may be utilized. It is important to carefully assess each patient and select the most appropriate surgical approach based on their individual needs and the characteristics of their condition. In 2013, Zhang et al. (5) reported a simple posterior debridement, bone graft, and internal fixation technique for treating upper thoracic spinal tuberculosis with kyphotic deformity. The authors believe that this procedure is suitable for cases with a small amount of abscess confined to the paravertebral area and with kyphotic deformity. The debridement can also be corrected using the pedicle screw technique, which offers advantages such as minimal trauma, good orthopedic outcomes, low hospitalization costs, and favorable treatment outcomes. However, some scholars express concerns that this approach may not completely eliminate abscess lesions and could potentially lead to the spread of tuberculosis and the development of drug resistance. Therefore, they advocate for anterior debridement. The anterior approach allows for the removal of abscesses and direct visualization for spinal stability reconstruction. If the posterolateral arc approach is adopted based on the lesion's severity, a “T”-shaped incision is made along the trapezius muscle. During the operation, one side of the rib joint and transverse process are removed to expose the pedicle and vertebral body. The pedicle is then removed to expose the dura mater, relieving the pressure on the spinal canal using a forearm retractor. This procedure focuses on posterior exposure and kyphosis correction, making it particularly advantageous for older lesions. Each surgical approach has its merits and considerations, and the choice of technique should be based on individual patient characteristics, lesion severity, and the surgeon's expertise.

However, Wang et al. (6) reported cases of esophageal injury resulting from the placement of long screws during the posterolateral approach for treating upper thoracic tuberculosis. The authors suggest that the utilization of intraoperative CT and surgical robot navigation can help mitigate such complications. These advanced technologies provide real-time imaging guidance and enhance surgical precision, thereby reducing the risk of iatrogenic injuries. The inclusion of intraoperative CT allows for detailed visualization of anatomical structures, enabling surgeons to navigate with greater accuracy and avoid vital structures such as the esophagus. Additionally, surgical robot navigation systems offer enhanced precision and stability during the placement of screws, minimizing the likelihood of inadvertent damage. By incorporating these technologies into the surgical procedure, surgeons can improve patient safety and optimize surgical outcomes in the treatment of upper thoracic tuberculosis. It is essential to continually explore innovative approaches and advancements in surgical techniques to enhance patient care and minimize potential complications. Cauchoix et al. (7) validated that sternotomy provides access to the anterior aspect of the upper thoracic spine and allows for full exposure of T4–T5 vertebral segment lesions. However, this approach is associated with significant surgical trauma, increased intraoperative bleeding, slow postoperative recovery, and a higher incidence of complications. Consequently, its widespread clinical adoption remains limited. In cases where upper thoracic tuberculosis necessitates comprehensive treatment, a combined anterior and posterior approach may be considered. This approach offers the theoretical advantage of more thorough lesion removal and the ability to achieve stable posterior column reconstruction. Nevertheless, it is important to acknowledge that this technique is associated with a longer operation time, greater intraoperative blood loss, prolonged bed rest, and extended hospitalization. Additionally, the risk of postoperative complications such as deep vein thrombosis increases. Therefore, careful consideration must be given to a patient's overall condition and their ability to tolerate surgery before opting for the combined approach. As the field of surgical interventions for upper thoracic tuberculosis continues to evolve, it is crucial to assess the benefits and drawbacks of different surgical approaches, taking into account patient-specific factors and optimizing treatment outcomes. Ongoing research and clinical practice will guide the selection of the most appropriate approach for each individual case, ensuring the best possible patient care.

Lessons can also be drawn from the treatment approaches used for upper thoracic fractures, dislocations, disc herniations, and tumors. The surgical procedure typically involves adopting a left lateral position with a right-sided approach. An arc-shaped incision is made along the inner and lower edges of the scapula, approximately 3 cm outside the spinous process of the affected vertebra, extending to the posterior axillary line. Sequentially, the incision involves cutting through the skin, subcutaneous tissue, lumbar dorsal fascia, a portion of the trapezius muscle, latissimus dorsi muscle, rhomboid muscle, and serratus anterior muscle. Additionally, the third rib is resected. Subsequently, vertebrectomy, bone graft fusion, and internal fixation are performed, yielding favorable outcomes. However, it should be noted that this approach necessitates extensive muscle dissection, which may impact postoperative rehabilitation. Although adopting this incision approach allows for effective treatment of upper thoracic conditions, the wide range of muscle dissection involved raises concerns regarding the patient's recovery and rehabilitation. Therefore, careful consideration should be given to balancing the benefits of the surgical approach with the potential impact on postoperative rehabilitation. Individual patient factors, including overall health and rehabilitation potential, should be taken into account when determining the most appropriate approach for upper thoracic conditions. Rossitti et al. (8) employed the surgical approach of an anterior cervical extended incision for treating upper thoracic (T1-T2) disc herniation, which has shown promising outcomes. However, the literature does not provide detailed information on the advantages and disadvantages of this approach compared to the third rib small incision approach. Further research and clinical studies are warranted to explore and evaluate the efficacy and potential drawbacks of these different surgical approaches. Rensnick et al. (9) obtained satisfactory outcomes in the treatment of 21 patients using the low anterior cervical approach. However, it is worth noting that this approach has certain limitations in terms of the extent of incision exposure, making it challenging to fully expose lesions located at the T3 and T4 levels. Further investigation and comparative studies are needed to assess the efficacy and feasibility of alternative approaches for addressing lesions in these specific areas. Yasui et al. (10) documented the treatment of ossification of the posterior longitudinal ligament (OPLL) at the cervicothoracic junction using a combined approach involving an inverted T-shaped split sternal stem and cervical approach. This surgical technique allows for exposure down to the T3 vertebral body, facilitating vertebral resection and removal of the ossified tissue. Luk et al. (11) conducted unilateral L-shaped or bilateral inverted T sternotomy in five patients, with no operation-related complications observed. This approach provides a broad exposure range, allowing direct visualization of lesions causing compression in front of the spinal cord and offering excellent maneuverability. However, reaching lesions below T4 is challenging due to the presence of the right atrium and ascending aorta, and sternal splitting may result in secondary injury, hindering early patient rehabilitation.

In cases of tumors affecting the upper thoracic vertebrae, the surgical approach should adhere to the principles of tumor exposure, complete removal, and the formulation of surgical strategies based on the tumor's location and extent of invasion. In situations involving upper thoracic tumors affecting the vertebral body and associated structures, posterior surgery can be considered as an appropriate approach. Cahill et al. (12) reported satisfactory outcomes with the use of combined anterior and posterior approaches, specifically total spondylectomy and posterior column fixation. Komagata et al. (13) employed an anterolateral approach to successfully remove T2–3 tumors, yielding favorable postoperative outcomes. Similarly, for tumors in the lower cervical spine, approaches such as the sternoclavicular joint approach and sternotomy approach have been utilized. However, it is worth noting that these approaches may present challenges in achieving optimal exposure of T4 and T5 vertebrae.

In conclusion, the approach discussed by the author for the treatment of upper thoracic tuberculosis through the third rib small incision approach has been relatively underreported in domestic literature. However, its applicability can be extended to the treatment of upper thoracic fractures, dislocations, and tumors. The following key points should be considered during the procedure: First, meticulous attention must be given to ligating the segmental vessels, and caution should be exercised when using electrocoagulation to avoid potential complications. Due to the negative pressure within the thoracic cavity, the combined effect of blood vessel pressure and negative thoracic pressure may result in post-electrocoagulation bleeding.

The advantages of this surgical approach are as follows: firstly, soft tissue injury: Dealing with upper thoracic lesions has been a challenge in clinical practice. The traditional anterior approach requires a long arc incision through the subscapular wall, necessitating the stripping of scapular muscles and pulling the scapula back. This results in a large incision and significant muscle damage. However, with this approach, there is no need to cut the latissimus dorsi muscle, expose the scapula, or cut the pectoralis major muscle. As a result, the incision length is significantly reduced, muscle injury is minimized, and shoulder motor function remains unaffected post-surgery. The senondly, exposure range: During the operation, the surgical site allows for excellent exposure of the thoracic 2–7 vertebral bodies. After pulling apart the aortic arch and heart, there are no obstructions from any tissue structures. This provides clear visibility, facilitates convenient operation, and enables the complete removal of the affected vertebral body. Thirdly, internal fixation: Another challenge in treating upper thoracic lesions is the difficulty in achieving internal fixation due to rib obstruction. However, with this approach, internal fixation can be smoothly performed on the thoracic 2 vertebral body. In cases where screws need to be implanted in the thoracic 1 vertebral body, the second rib can obstruct the use of a straight cone, requiring the screws to be tilted into the thoracic 1 vertebral body. Overall, these advantages contribute to the effectiveness of this approach for addressing upper thoracic lesions.

Due to the relatively low incidence of upper thoracic lesions and the complexity of the surgical approach, this method is considered as one of the viable options for managing such cases.

## Data Availability

The raw data supporting the conclusions of this article will be made available by the authors, without undue reservation.
